# Transmission of Respiratory Syncytial Virus genotypes in Cali, Colombia

**DOI:** 10.1111/irv.12833

**Published:** 2021-04-08

**Authors:** Maria Aurora Londono‐Avendano, Melissa Peláez‐Moreno, Eduardo López Medina, Mabel Soraya Moreno Turriago, Beatriz Parra Patiño

**Affiliations:** ^1^ Virus and Emerging Diseases ‐VIREM, Department of Microbiology College of Health, Universidad del Valle Cali Colombia; ^2^ Currently at Public Health Secretariat Caquetá Colombia; ^3^ Pediatric Infectology Study Center (CEIP); ^4^ Department of Pediatrics College of Health, Universidad del Valle Cali Colombia; ^5^ Clínica Imbanaco Cali Colombia; ^6^ Municipal Public Health Secretariat, Santiago de Cali Cali Colombia; ^7^ Currently at epidemiology COOMEVA EPS Cali Colombia

**Keywords:** Cali, Colombia, genotype, glycoprotein G, respiratory syncytial virus

## Abstract

**Background:**

Colombia's climatological variety, added to pathogen diversity, creates local niches for infectious diseases. In Bogotá, respiratory syncytial virus causes 30%‐52% of the cases of respiratory infections. In coastal or inter‐Andean cities with higher temperature and longer dry seasons, frequency of this virus is 7%‐13%. By 2017, increased hospitalizations due to airway infections occurred in regions whose weather is differently influenced by “El Niño Southern Oscillation” than in Bogotá, although microbial diversity might have also been involved.

**Methods:**

For Cali, an inter‐Andean city with warm tropical weather, records of respiratory syncytial virus from 2014 to 2018, in children two years old or younger, were analyzed, and genotypes transmitted during 2016‐2017 were identified based on partial sequences of glycoprotein G.

**Results:**

Most cases of respiratory syncytial virus in Cali occur in the first semesters, with peaks expressed around March‐April, without a clear association with pluviosity. Unlike the biannual rotating pattern of Bogotá, co‐circulation of types A and B was detected. As years pass, transmission seasons are becoming longer and frequencies of the virus augment. The viral genotypes identified follow international trends with dominance of Ontario and Buenos Aires clades. Similar to other isolates in these clades, viruses from Cali exhibit glycosylation variability that may account for their fitness.

**Conclusions:**

The pattern of respiratory syncytial virus transmission in Cali differs from that in Bogotá. Its epidemiology is shifting and will remain so with the advent of novel respiratory diseases. This may impact the introduction of vaccination schemes for these or other respiratory viruses.

## 1 INTRODUCTION

Respiratory syncytial virus (RSV) is one of the main causes of acute respiratory infection (ARI) among infants and the elderly. This virus is particularly associated to the admission into intensive care units in children under the age of two years, who develop either influenza‐like illness (ILI) or severe acute respiratory infection (SARI).^1^, ^2^, ^3^ Environments with low humidity and high temperatures favor the survival of RSV virions, while during the winter or rainy seasons, the number of infections increases, since more people remain indoors.^4^, ^5^ In temperate zones, this virus has seasonal transmission with onsets matching the coldest weeks of winter.^1^, ^6^, ^7^ In equatorial countries, RSV is involved in ARI cases all year round, with outbreaks in those rainy seasons of high humidity and low temperature.

RSV has been classified in two types (RSV‐A and RSV‐B), based on genetic differences in the region encoding glycoprotein G, a protein that facilitates binding of the virus to epithelial cells of the airway.^7^ Within these types, 11 RSV‐A (GA1‐GA7, NA, SAA, ON, LBA) and 23 RSV‐B genotypes (GB1‐GB4, SAB1‐SAB4, URU1, URU2, BA1‐BA12, THB) have been established.^9^, ^10^ New RSV genotypes arise by mutations and duplications in the glycoprotein G gene and spread according to their antigenicity and virulence; these properties are also influenced by substitutions in the fusion protein.^8^, ^10^, ^11^

In Colombia, RSV is endemically transmitted throughout the year, with higher occurrence during the rainy seasons.^12^, ^13^, ^14^ RSV‐A and RSV‐B circulate in a biannual rotational dynamic characterized by an increase in cases and hospitalizations when RSV‐A predominates.^15^ This observation results from a couple of studies and whether the prediction accommodates to demographics and climatic diversity across the country is yet to be established.^12^, ^14^, ^16^ Recently, 37 samples collected between 2000 and 2009 were genotyped for RSV.^17^ Researchers found genotypes RSV‐A‐GA2, RSV‐A‐GA5, and RSV‐B‐BA circulating in the country; however, data are scattered at random dates within this period, and most samples come from a single location.

New variants of RSV‐A with duplications in glycoprotein G are replacing genotypes like those found in Colombia by Avila and coworkers. Among these, RSV‐A‐NA1 was associated with severe bronchiolitis in children,^18^ and RSV‐A‐ON1 has been linked to higher severity of ILI, to a sooner onset for hospitalization,^19^ and to longer stays in intensive care units.^20^ Genotype RSV‐A‐ON, initially detected in Ontario (Canada) in 2010, is believed to have virulence comparable to its more likely predecessors, genotypes RSV‐A‐GA2 and RSV‐A‐NA1,^19^, ^21^, ^22^ but with a higher dispersal capacity.^23^, ^24^ In spite of its broad distribution during recent years, no official record has been established on the presence of genotype RSV‐A‐ON in Colombia.

According to the National Epidemiological Surveillance System (SIVIGILA), onsets of RSV transmission in Cali, main city in Valle del Cauca, coincide with those reported nationwide.^13^, ^25^ However, for 2016 and 2017, nationwide proportions of RSV in virus‐positive samples were 51% and 67%, while in Cali the RSV percentages among virus positives were 50% and 51%.^26^, ^27^ In 2017, increase of ILI‐SARI and augment of RSV and influenza A frequencies was observed in Valle del Cauca.^25^ Although many of these differences are attributable to methodological variations in RSV detection, microclimatic and virus‐derived factors may also account for RSV prevalence across the Colombian territory. This work deepens the analysis of the epidemiology of ILI‐SARI cases associated with RSV among infants in Cali, by reviewing the virus circulation patterns in 2014 ‐ 2018, and detecting genetic variants circulating in the city in years 2016‐2017.

## 2 METHODS

### 2.1 ARI cases related to RSV in Cali

To construct a RSV circulation curve in children in Cali, younger than two years of age, information contained in *SIVIGILA*, from 2014 to 2018, was analyzed. Data were provided by collaborators in charge, according to protocols for data protection. Cases of ILI are those of acute respiratory infections with fever ≥ 38°C and coughing symptoms of maximum seven days of evolution, requiring ambulatory care; SARI cases include patients with acute respiratory infections with a history of fever and coughing not longer than 10 days, who require hospitalization. Indirect immunofluorescence (IFI) is used for virus detection, as guided by Colombian Institute of Health. From 2014 to 2018, a total of 6,440 records were obtained for the region, 2,148 of them meeting the criteria for this study.

### 2.2 Samples for RSV genotyping

Specimens come from the biobank of respiratory samples processed in Laboratory for Diagnosis of Biological Agents (LDAB) at Universidad del Valle, from 2016 to 2018. Typification was done to samples randomly selected among the RSV positives, in children younger than two, from 2016 to 2017. Procedures had the approval number 015‐016 from the institutional human research ethics committee of College of Health from Universidad del Valle. For a subset of specimens, information on clinical evolution of the child was provided by the medical team. Screening for respiratory viruses in these samples was performed by IFI with the “Respiratory panel viral screening and identification IFA Kit,” (Light Diagnostics™; cat. # 3105*)* and “Human metapneumovirus (hMPV) direct immunofluorescence assay" (Light Diagnostics™; cat. Cat. # 3124) (Millipore Corporation, Livingston, UK). These kits detect influenza A, influenza B, RSV, adenovirus, parainfluenza 1, 2, and 3 and hMPV, by detecting viral antigens in infected cells. The immune preparations were analyzed under a fluorescence microscope using Evan's blue dye as contrast stain.

### 2.3 RSV subtype identification

The type of virus was identified by amplification of the region encoding glycoprotein G by multiplex RT‐PCR. Viral RNA purified with viral RNA minikit kit (Qiagen, Germantown, MD, USA). Amplification was performed with the SuperScript III One‐Step RT‐PCR system (Life Technologies Corporation, Carlsbad, CA, USA) and primers set G1A/G2A for RSV‐A (5'‐TCAAGCAAATTCTGGCCTTA‐3 '/ 5'‐CTGCAATTCTGTTACAGCAT‐3')^28^ and G1B/BGR for RSV‐B (5'‐ACAAGCAAATTTTGGCCCTA‐3 '/ 5'‐TGCCCCAGRTTTAATTTCGTTC‐3').^28^, ^33^ These pairs of oligonucleotides generate bands of approximately 1,600 bp in RSV‐A and 1,400 bp in RSV‐B, which makes it possible to differentiate RSV types and identify dual infections using 1.5% agarose gels.

### 2.4 Genotype assignment and variability in selected samples

The hypervariable region, a fragment of about 270 nucleotides for RSV‐A and 360 for RSV‐B, which encodes the second mucin‐rich region,^29^ was used for taxonomic and phylogenetic characterization. Amplicons were cleaned directly or using agarose gels (NucleoSpin^®^ Gel and PCR Clean‐up, Macherey‐Nagel, Switzerland) and sequenced by the Sanger method with primer AL18 (5'‐TTGGCAATGATAATCTCAACYTC‐3 ') plus either primer G2A or BGR; primer AL18 was designed to bind in the central conserved domain of the glycoprotein G in all subtypes of RSV. Sequences were assembled by mapping to a reference in Geneious 6.1.6 [Biomatters, Newark, USA]. The resulting contigs were aligned by ClustalW to reconstruct phylogenies with the maximum likelihood method in MEGA 7.0.^30^ Reference sequences for the 11 recognized genotypes of RSV‐A and representatives of main genotypes of RSV‐B were included. Potential for N‐glycosylation was assigned to residues scoring more than 0.5 in at least two of predictors NetNglyc1.0, Glycomine and N‐GlyDE^31^; potential for O‐glycosylation was evaluated with Glycomine, as recommended.^32^

### 2.5 Relationship between RSV subtype and symptoms

The association between the main clinical manifestations and RSV type was analyzed by comparing proportions between independent groups. Qualitative variables (fever, coughing, etc) were compared using Fisher's exact test with Bonferroni correction. Student's test was used for the comparison of quantitative data (ie, length of stay in intensive care unit); values were taken as different when the corresponding test showed a probability value lower than 0.05.

## 3 RESULTS

### 3.1 Surveillance of RSV infections in Cali

**FIGURE 1:**
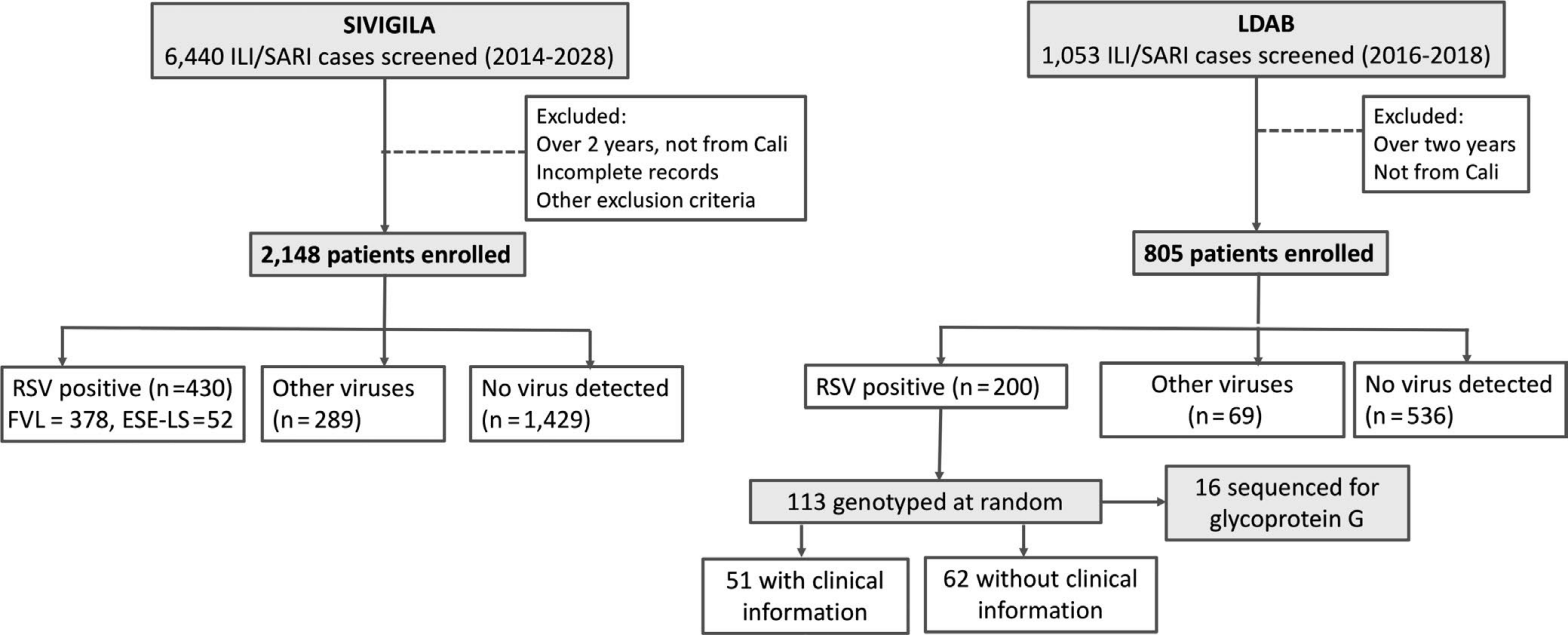
**Study design**. To describe circulation of RSV in children in Cali, records of influenza‐like illness or severe acute respiratory infection fed to SIVIGILA, from two sentinel centers, were filtered and analyzed. To identify variability of RSV, samples from children younger than two, submitted to the LDAB service in 2016‐2018 for a respiratory virus test, were used. A proportion was typified to identify RSV‐A and/or RSV‐B; subsequent analysis in LDAB data subsets included the following: a) genotypification in 14% of typified samples by sequencing the hypervariable region of glycoproteins G; b) scrutiny of medical records to evaluate the effect of RSV variability in symptomatology

Surveillance for ILI‐SARI in the city has improved during the last five years, with the consequent augment in RSV reports. By 2014, these data were almost absent, but in the following years reports increased by four to eightfold. Between 2014 and 2018, a total of 6,440 ILI‐SARI cases were reported to SIVIGILA from Valle del Cauca, 4,083 of them autochthonous from Cali (Figure 1). Almost half of these cases, 52.61% (2,148), corresponded to children two years of age, or younger, RSV occurred in 20.02% of the 2,148 minors, 13.5% were positive for other viruses, and 66.5% of them did not have a viral infection. RSV frequency in these children was 23.5, 17.2, 18.4, 19.6, and 20.1, for 2014, 2015, 2016, 2017, and 2018, respectively (Supplementary Table 1). Most RSV positives were reported from FVL, a facility that serves communities with higher income as compared to ESE‐LS.

**FIGURE 2:**
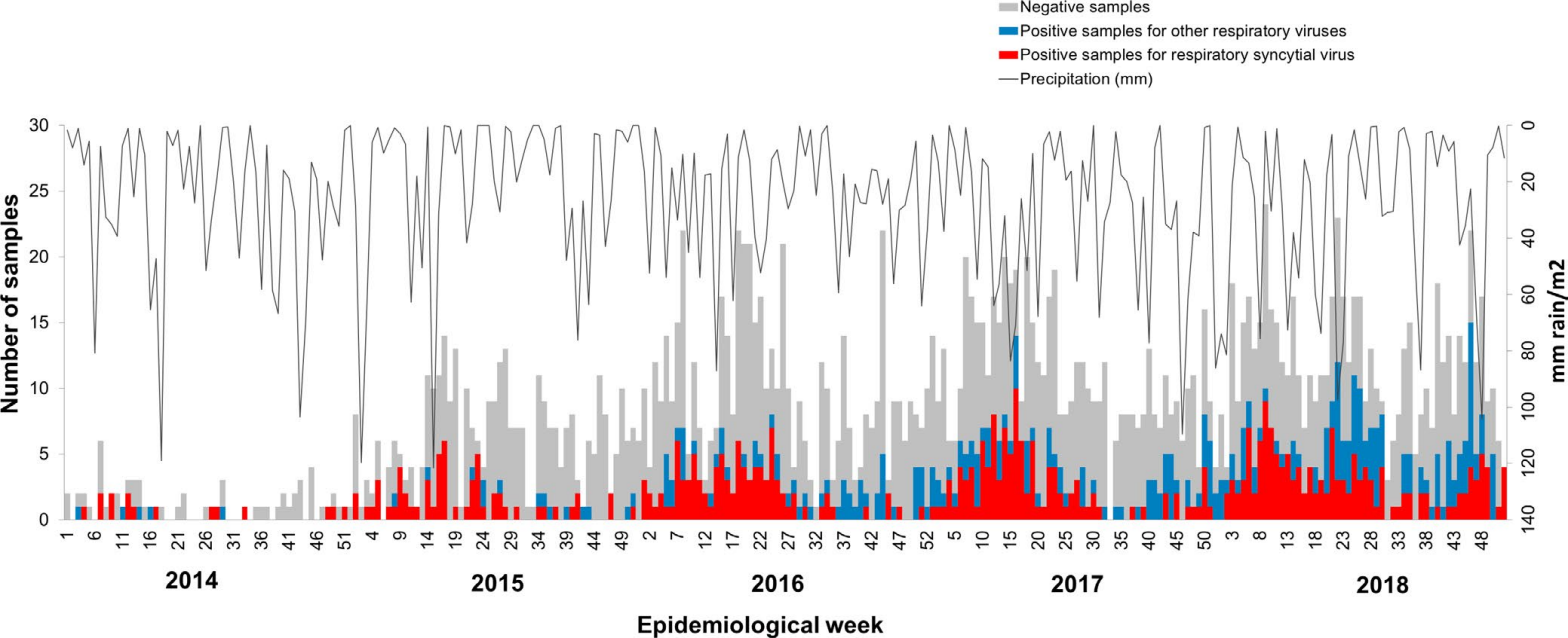
**RSV dynamics in the surveillance dataset from Cali (2014‐2018).** Data include autochthonous ARI cases from Cali, in children two years of age, or younger. Left y‐axis indicates samples identified as RSV positives (red bars), having another respiratory virus (blue bars), or negative for the seven viruses routinely screened (gray bars). A black line shows the average precipitation per epidemiological week, with values indicated in the right y‐axis

In children under two years, the male to female ratio of RSV infections was almost equal during all years (1:2.2, 1.45:1, 1:1.04, 1.26:1, and 1:1.08 for 2014, 2015, 2016, 2017, and 2018, respectively) (Supplementary Table 1). Average age in the ILI‐SARI cases was 9.8 months, but 8.6 months was the average age for RSV positives. Among the 1,053 samples received at LDAB, 805 corresponded to children two years of age, or younger, with an average age of 7.2 months; the minors were predominantly males, with a male to female ratio of 1.9:1 among RSV positives; proportion of RSV among virus positives in here were 84.6%, 69.5%, and 78.6% in 2016, 2017, and 2018, respectively (Figure 2, Supplementary Table 1).

Onsets of RSV transmission in Cali match those periods with higher precipitation during the first semester of 2015, 2016, 2017, and 2018, although the more intense rainy seasons not necessarily correlate with a higher RSV transmission (see second semester results in 2014 and 2017, in Figure 2). Improvement in ARI surveillance is one of the reasons which may explain these results. While RVS cases drastically diminished after week 25 in 2014, 2015, and 2016, this reduction is less evident in 2017 and basically disappears by 2018.

### 3.2 Distribution of RSV‐A and RSV‐B in LDAB samples

**FIGURE 3:**
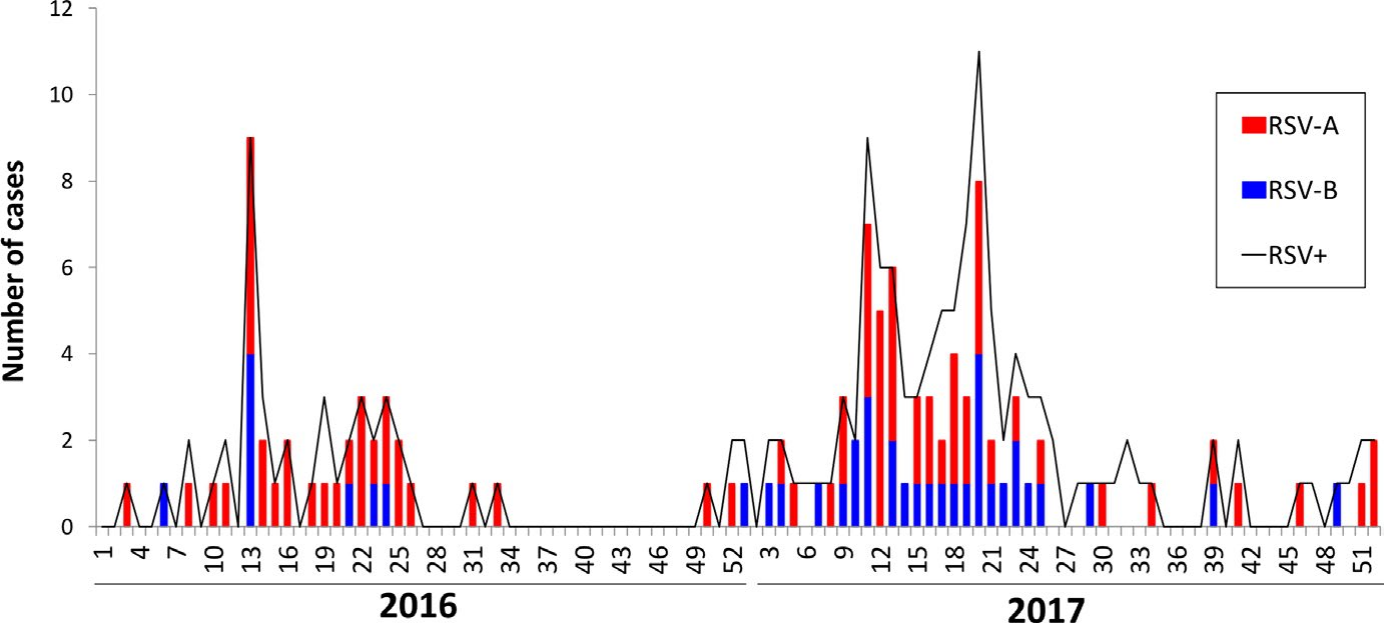
**Distribution of RSV cases identified in samples processed in LDAB, in 2016 and 2017**. The black line indicates the overall number of RSV cases detected per week; bars show the number of samples typified as RSV‐A (red) or RSV‐B (blue) in randomly selected weeks

During 2016, the majority of RSV positives occurred between epidemiological weeks 13 ‐ 25. In the first semester of 2017, there was an increase in cases of RSV between weeks 11 ‐ 13 (see Figure 3), but a second and more conspicuous onset was detected in weeks 19‐21, two weeks behind the main transmission peak of RSV in SIVIGILA data for 2017 (Figure 2). Although samples received at LDAB in 2018 were not subject to RSV typification, peaks of cases occurred during weeks 8‐12, with weeks 14 and 21 having the highest rate of RSV positives (data not shown).

RSV‐A was detected in 77% (30/39) of the samples, almost every week of 2016, while RSV‐B appeared only in a few weeks, scattered across the first semester of this year. For 2017, frequency of RSV‐A dropped to 58% (43/74) and distribution of RVS‐B extended to many weeks in both semesters. Hence, co‐circulation of the two types of RSV was detected (Figure 3) with RSV‐A predominating at the beginning but the proportion of the two types was balanced during most of 2017. Neither type can be blamed for the abrupt increase in RSV recorded in week 13 of 2016, nor for the peaks of highest transmission occurring in weeks 11 and 20 of 2017 (Figure 3).

### 3.3 RSV‐A and RSV‐B genotypes in Cali 2016 ‐ 2017

**FIGURE 4:**
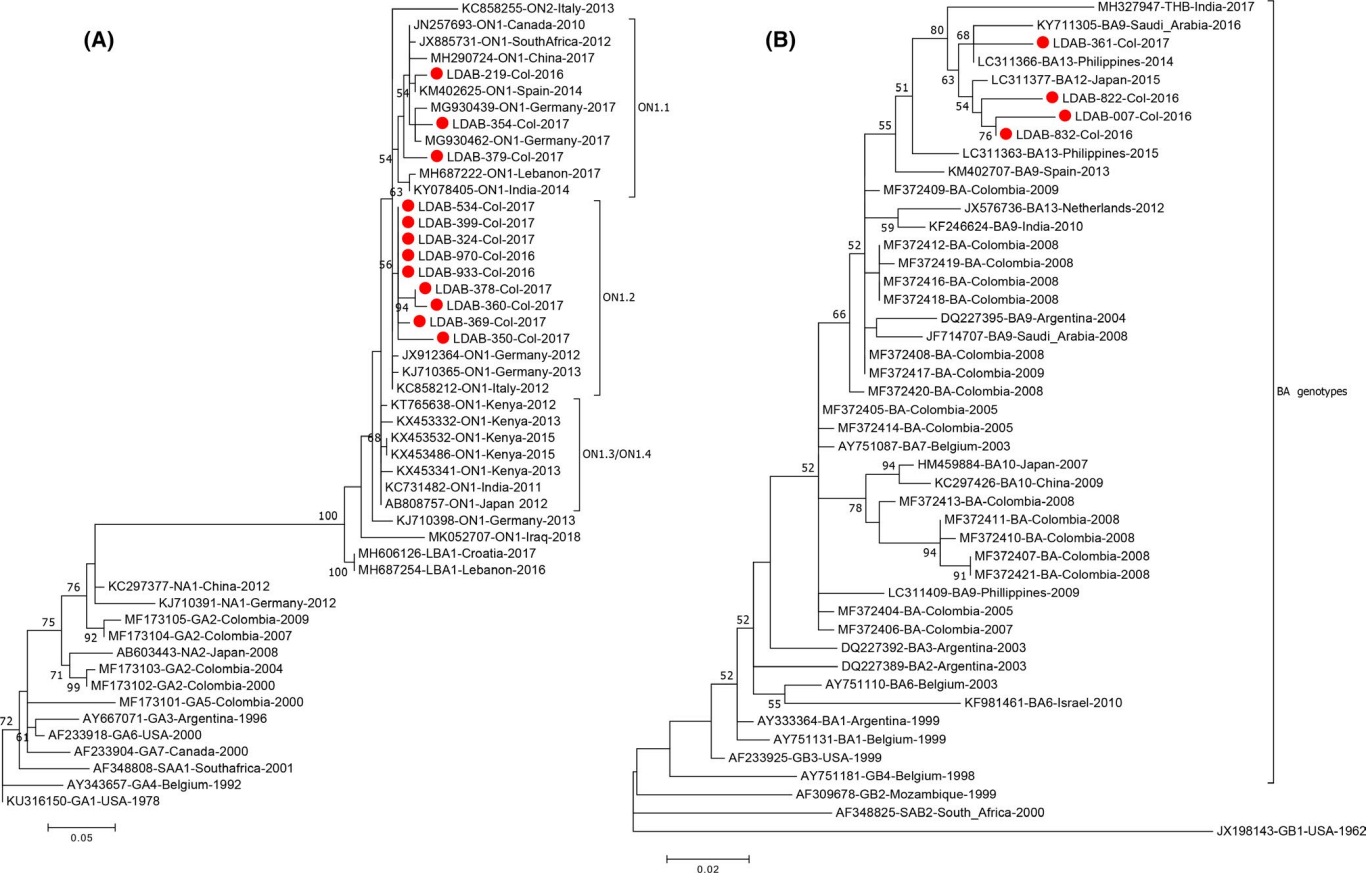
**Clustering of RSV‐A and RSV‐B specimens from Cali**. (A) Phylogenetic grouping of the hypervariable region of RSV‐A compared to reference sequences of ancient and novel RSV‐A genotypes, plus an extended view of ON lineage. (B) Phylogenetic reconstruction of hypervariable region of RSV‐B, showing clusters of BA genotypes. Sequences coming from this work are indicated with red dots. Both phylogenies were constructed in MEGA7 using the maximum likelihood method based on the general time reversible model and considering all nucleotides as informative; the unit for branch lengths is number of nucleotide substitutions per site 30

Genotype of RSV‐A detected during both years was ON, while RSV‐B sequences belong to the BA clade (see Figure 4A and 4B). Within the ON major clade, two minor clades, the ON1 and ON2, have been proposed. ON1 can be subdivided into at least four subclades (ON1.1, ON1.2, ON1.3, and ON1.4)^23^, ^33^ and sequences from Cali fit the ON1.1 and ON1.2 clades, but no representatives of the ON1.3 were found. Similarly, BA genotype of RSV‐B has evolved into multiple subclades, including BA1 to BA10, plus additional groups recently proposed.^9^, ^10^ Sequences from Cali collapsed into a subclade containing representatives of BA lineages classified by diverse authors as BA9, BA12, BA13, or even TBH, according to their respective approach; the isolates analyzed here correspond to the newest genotypes of RSV‐B and have probably diverged from those previously found circulating in Colombia.^17^

### 3.4 Amino acid profile in the hypervariable region of glycoprotein G

**FIGURE 5:**
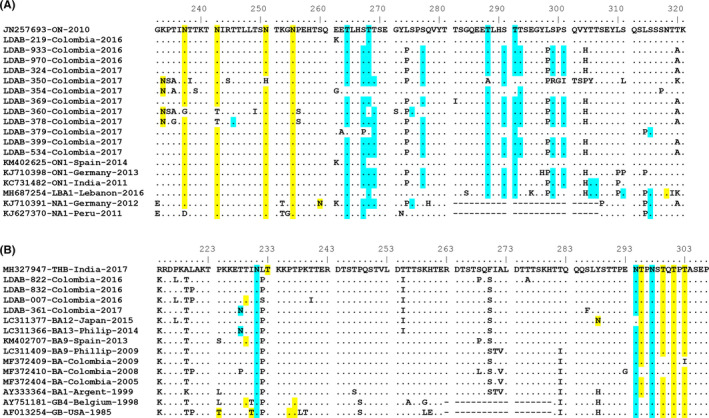
**Analysis of glycosylation potential in the second mucin‐rich domain of glycoprotein G**. (A) Alignment of residues 232‐321 in RSV‐A isolates from Cali compared to reference sequences of ON, LBA1, and NA1 clades. (B) Alignment of amino acids 214‐307 of RSV‐B isolates from Cali compared to reference sequences of BA and GB clades. Sequences were aligned by MUSCLE in MEGA7 and manually edited. Residues shadowed in yellow and blue are those with a predicted potential of N‐glycosylation and O‐glycosylation higher than 0.5, respectively

As observed in Figure 5A, the fragment of glycoprotein G sequenced for these samples revealed the duplication characteristic of ON variants (residues 282‐306). There were 12 variable sites compared to five in the original region (residues 259‐289). However, the variation per site is low, as ten of the variable positions detected in the duplication are unique to one sequence. Residues prone to be N‐glycosylated occur before this duplication, and four of them (N237, N242, N251, and N255) are highly conserved among isolates from Cali, ON variants and representatives of NA1 genotype; an additional site with potential for N‐glycosylation occurs in a few isolates from Cali. Amino acids with potential for O‐glycosylation are placed near or in the duplication itself; except for LDAB‐369, which has two of these residues, isolates from Cali have four or more residues that cross the threshold for O‐glycosylation potential, similar to KJ710391‐NA1 and the ON variants referenced.

Alignment in Figure 5B shows the duplication characteristic of the BA clade and how it is involved in the diversification of glycoprotein G. All clades contain conserved sites for O‐glycosylation at the c‐terminal, while N‐glycosylation sites, also conserved, are located before and after the duplicated region. Interestingly, the duplicated region in these variants is not accumulating residues prone to glycosylation and variability is low, if compared to the duplicated stretch in RSV‐A variants.

### 3.5 RSV genotype and clinical manifestations

About one third of samples positive for RSV correspond to cases whose medical records were available (Supplementary Table 1). Overall, RSV is causing mild to moderate infections that manifest with cough, nasal congestion, and respiratory difficulty. Hospitalization was required in 75% of these cases. Males were slightly more prone to RSV‐A (odds ratio 1.7, 95% CI 0.50 ‐ 5.73). In spite of the fact that the most common viral type in this subgroup is RSV‐A, comparison of proportions indicates that the viral type is not related to a greater severity of symptoms; children infected by RSV‐A tend to be younger and to need oxygen for a longer period of time. However, p‐values do not support these periods of time as different clinical behaviors (Supplementary Table 2).

## 4 DISCUSSION

RSV transmission pattern in Cali is differentiable from that described in national conglomerates and data exclusively from Bogotá, although some characteristics of the main transmission season are common. In Bogotá, where historically more data are captured by SIVIGILA,^34^ RSV prevalence ranges between 30% and 52%, with onsets correlated with the first rainy season, during the months of March‐May.^16^ Bogotá has an average temperature of 13.1°C and relative humidity ranging from 77% to 83%, whereas some other main cities in the country are coastal or inter‐Andean, with average temperatures above 20°C and longer dry seasons. Prevalence of RSV in the warmest cities ranges between 7% and 13%, without a clear link to rainy seasons.^16^

According to oceanic Niño indexes, a variable that uses changes of oceanic temperature in the Pacific area influenced by El Niño Southern Oscillation to define warm (“el niño”) and cold (“la niña”) intervals, the most recent warm period was recorded for 2016, followed by a subtle decrease of temperatures in 2017 and 2018^36^; 2014 and 2015 were also cold periods in the Niño1 + 2 sector in which the Colombian Pacific is located. Rainy patterns for 2014 and 2015 allow to differentiate dry seasons, here taken as those with precipitation lower than 40 mm (Figure 1). However, higher precipitation seems to be becoming more frequent and less seasonal, which may explain the continued transmission of RSV observed in 2018 and, in part, the higher record of ARI cases in 2016, 2017, and 2018.

The pattern of transmission of RSV observed in Cali is co‐circulation of the two virus types, which implies that infants are at risk of reinfection.^35^ However, no cases of co‐infection with multiple genotypes were observed. Putting together SIVIGILA data with RSV types detected per week, the trend is to have longer transmission seasons of both genotypes. Whether this is an effect of the above‐mentioned climatological phenomenon could be evaluated by expanding RSV variability surveillance. Evidence accumulated here suggests that in Cali, RSV transmission seasons are changing to last all year round.

RSV‐A samples characterized clearly belong to the contemporary ON clades.^23^, ^33^ The same was obtained for RSV‐B, with variants that group to the BA9 and more recent subclades.^9^ This variability has been described in different parts of the world, and therefore, in Cali the transmission of RSV does not occur as an isolated event, but rather as a result of the successful genotypes spreading throughout all continents.

We found no association of the type of virus with disease severity. However, findings are to be taken cautiously as the small number of samples analyzed here could be misleading. Similarly, associations between RSV‐A‐ON and SARI, as previously reported,^19^, ^20^, ^24^ could not be evaluated with the amount of genetic variation obtained in this study.

Since glycoprotein G is one of the main antigens on the surface of RSV, its variability is directly reflected in viral fitness. In the second hypervariable region, known to support a considerable amount of carbohydrates,^37^ the glycosylation potential showed no particular glycosylation profile as a characteristic of the newest RSV variants. However, a series of conserved threonine O‐glycosylation sites were spotted as likely indispensable for the functioning of this domain. The duplicated region of RSV‐A augments the amount of N‐glycosylation sites, but this is not the case in RSV‐B variants. In RSV‐B variants, the duplication increased the distance between conserved glycosylation sites; whether this has structural effects, such as an improvement in the affinity of glycoprotein to membranes, or an evasion of immune response, is yet to be established.

This work represents an update of RSV epidemiology in Colombia and describes, for the first time, the circulation of ON1 genotype in the country. Unlike samples collected from Bogotá and a few other places before 2010, only RSV genotypes with duplications of glycoprotein G were found in Cali, suggesting a complete establishment of these genotypes in the country. There was no association between RSV type or genotype to the severity of SARI, which may also indicate that clinical manifestations depend on patient's risk factors that promote RSV infection complications.

## CONFLICT OF INTEREST

We declare no conflict of interest.

## Supporting information

TableS1‐S2
