# Supplementary material for: Transmission of Respiratory Syncytial Virus genotypes in Cali, Colombia
Source: Influenza Other Respir Viruses. 2021 Apr 8;15(4):521–8. doi: 10.1111/irv.12833 (PMC8189202; doi:10.1111/irv.12833)
Supplement: Supplementary file 1 — TableS1‐S2 [file IRV-15-521-s001.docx]

**Supplementary Table 1. Frequency of RSV in datasets analyzed for Cali**

|  | **SIVIGILA CALI** | | | | | **LDAB** | | |
| --- | --- | --- | --- | --- | --- | --- | --- | --- |
|  | **2014** | **2015** | **2016** | **2017** | **2018** | **2016** | **2017** | **2018** |
| Total < 2 y.o. | 68 | 335 | 521 | 578 | 646 | 190 | 465 | 150 |
| Av. age (years) | 0.93 | 0.84 | 0.93 | 0.92 | 0.49 | 0.61 | 0.59 | 0.56 |
| Male | 33 | 179 | 275 | 336 | 330 | 119 | 279 | 102 |
| IFI results (in percentage) | | | | | | | | |
| RSV | 23.5 (16/68) | 17.6  (59/335) | 18.5  (96/521) | 19.5  (113/578) | 22.6  (146/646) | 23.2  (44/190) | 24.1  (112/465) | 29.3  (44/150) |
| Other viruses | 7.4  (5/68) | 4.2  (14/335) | 10.7  (56/521) | 14.2  (82/578) | 20.4  (132/646) | 4.2  (8/190) | 10.6  (49/465) | 8.0  (12/150) |
| Negative | 69.1  (47/68) | 78.2  (262/335) | 70.8  (369/521) | 66.3  (383/578) | 57.0  (368/646) | 72.6  (138/190) | 65.3  (304/465) | 62.6  (94/150) |

**Supplementary Table 2. Epidemiological features in patients for which a clinical follow up was available**

| **Samples** | **RSV-A** | **RSV-B** | **P** |
| --- | --- | --- | --- |
| Patients | 33 | 18 | --- |
| Median age, months ± STDV | 5.75(5.95) | 4.31(4.88) | 0.38^‡^ |
| Males | 24 | 11 | 0.29* |
| Onset to consultation, days ± STDV | 4.79(3.13) | 4.28(2.49) | 0.56^‡^ |
| Symptoms |  |  |  |
| Cough | 28 | 18 | 0.15* |
| Fever | 15 | 12 | 0.24* |
| Runny nose | 23 | 13 | 0.88* |
| Breathing difficulty | 19 | 15 | 0.12* |
| Disease severity |  |  |  |
| Low | 10 | 6 | 0.79* |
| Mild | 12 | 6 | 0.92* |
| Severe | 7 | 5 | 0.74* |
| Health care |  |  |  |
| Hospitalization | 24 | 14 | 0.75* |
| ICU | 6 | 2 | 0.71* |
| Mean stay in ICU, days ± STDV | 2.08(4.82) | 0.69(1.72) | 0.32^‡^ |
| Need for oxygen | 21 | 11 | 0.88* |
| Time with oxygen, days ± STDV | 4.7(5.82) | 3.31(3,25) | 0.43^‡^ |

^‡^ Student’s t test

*Chi-square with Yates correction or Fisher two-tailed test if values < 0.05.
